# Data for molecular dynamics simulations of *Escherichia coli* cytochrome *bd* oxidase with the Amber force field

**DOI:** 10.1016/j.dib.2021.107401

**Published:** 2021-09-23

**Authors:** Surl-Hee Ahn, Christian Seitz, Vinícius Wilian D. Cruzeiro, J. Andrew McCammon, Andreas W. Götz

**Affiliations:** a Department of Chemistry and Biochemistry, University of California San Diego, 9500 Gilman Drive, La Jolla, CA 92093, United States; b San Diego Supercomputer Center, University of California San Diego, 9500 Gilman Drive, La Jolla, CA 92093, United States; c Department of Pharmacology, University of California San Diego, 9500 Gilman Drive, La Jolla, CA 92093, United States

**Keywords:** Cytochrome bd oxidase, Tuberculosis, Molecular dynamics, Amber force field

## Abstract

Cytochrome *bd*-type quinol oxidase is an important metalloenzyme that allows many bacteria to survive in low oxygen conditions. Since *bd* oxidase is found in many prokaryotes but not in eukaryotes, it has emerged as a promising bacterial drug target. Examples of organisms containing *bd* oxidases include the *Mycobacterium tuberculosis (Mtb)* bacterium that causes tuberculosis (TB) in humans, the *Vibrio cholerae* bacterium that causes cholera, the *Pseudomonas aeruginosa* bacterium that contributes to antibiotic resistance and sepsis, and the *Campylobacter jejuni* bacterium that causes food poisoning. *Escherichia coli (E. coli)* is another organism exhibiting the cytochrome *bd* oxidase. Since it has the highest sequence identity to *Mtb* (36%) and we are ultimately interested in finding drug targets for TB, we have built parameters for the *E. coli bd* oxidase (Protein Data Bank ID number: 6RKO) that are compatible with the all-atom Amber ff14SB force field for molecular dynamics (MD) simulations. Specifically, we built parameters for the three heme cofactors present in all species of bacterial cytochrome *bd*-type oxidases (heme b${}_{558}$, heme b${}_{595}$, and heme d) along with their axial ligands. This data report includes the parameter and library files that can be used with Amber’s LEaP program to generate input files for MD simulations using the Amber software package. We also provide the PDB data files of the initial model both by itself and solvated with TIP3P water molecules and counterions.

## Specifications Table

**Table:** 

Subject	Chemistry, Biology
Specific subject area	Computational Chemistry, Biophysics
Type of data	Amber OFF library file, Amber parameter files, PDB files, XYZ files
How data were acquired	Density functional theory calculations with Gaussian 16; model preparation with Maestro (Schrödinger), CHARMM-GUI membrane builder, AmberTools, and MCPB.py; molecular dynamics simulations with Amber20
Data format	Raw PDB coordinate files, Raw XYZ coordinate files, Raw Amber OFF library file, and Raw Amber parameter files
Parameters for data collection	Starting geometries based on the cryo-EM structure from Protein Data Bank ID number: 6RKO
Description of data collection	Geometry optimizations, frequency calculations, and electrostatic potential (ESP) charge derivation of the three heme cofactors with axial amino acid ligands with BP86 and B3LYP functionals with empirical dispersion; molecular dynamics minimization, heating, and equilibration with Amber ff14SB, Generalized Amber force field (GAFF), Lipid 14 force field, TIP3P water molecules, Joung/Cheatham counterions, and new parameters for the heme cofactors
Data source location	University of California San Diego, La Jolla, CA, USA
Data accessibility	Data are within this article and available on https://doi.org/10.5281/zenodo.4742814[1]

## Value of the Data

• We report new force field parameters for the three heme cofactors present in all cytochrome bd-type oxidases, including heme d, which has not been previously reported in the literature.
• The new parameters will be useful to investigate cytochrome *bd* oxidase using molecular dynamics (MD) simulations.
• By using the structural ensembles of *bd* oxidase obtained from MD simulations, we can carry out virtual screening for inhibitors, which could potentially lead to new drugs for a variety of bacteria, such as turberculosis (TB), cholera, *Pseudomonas aeruginosa, Campylobacter jejuni* and other prokaryotes.

## 1 Data Description

The data provided in this article can be used for running molecular dynamics (MD) simulations of *Escherichia coli (E. coli)* cytochrome *bd* oxidase (Protein Data Bank ID number: 6RKO) [2]. The structure provided in this publication in the PDB file format (6rko_final.pdb, protein.pdb, protein.rst7) has been modelled to contain some of the missing residues in the experimental cryo-EM structure (Protein Data Bank ID number: 6RKO). Specifically, residues 240–248 and 334–339 in chain A were modelled with the Maestro software from Schrödinger [3]. Also, missing atoms in the palmitoyl-oleoyl-phosphatidylcholine (POPC) lipids were added back with the aid of the CHARMM-GUI membrane builder [4]. The structure of this system after solvation with TIP3P water molecules and counterions is also provided (protein_solv.pdb, protein_solv.rst7), as well as the structure of this system after minimization, heating and equilibration (protein_solv_equil.pdb, protein_solv_equil.rst7). All library and parameter files compatible with the Amber ff14SB force field needed to generate this system are also available (heme.lib, heme.frcmod, mcpbpy.frcmod, ubiquinone.prepi, ubiquinone.frcmod), along with the Amber LEaP script (tleap.in) that builds the complete system, yielding the parameter/topology and coordinate files for molecular dynamics simulations with Amber (protein.frcmod, protein_solv.frcmod). Finally, the optimized geometry of the three heme cofactors are provided in the XYZ file format (heme_b558.xyz, heme_b595.xyz, and heme_d.xyz). The repository includes all these data files and no other additional files are included.

## 2 Experimental Design, Materials and Methods

### 2.1 Model preparation (PDB data file generation)

The initial atomic coordinates were taken from the Protein Data Bank ID number: 6RKO [2], which is the 2.7 Å resolution cryo-EM structure of *Escherichia coli (E. coli)* cytochrome *bd* oxidase. Using the Maestro software from Schrödinger [3], the hydrogens were added to the residues, and the protonation states of the residues were assigned using PROPKA [5] at pH 7.5, same as in Ref. [2]. The placement of the hydrogens for the heme cofactors and the protonation states of several residues referred to in Ref. [2], which are residues nearby the heme cofactors that are involved in the proton and oxygen pathways, were matched to Ref. [2]. The positions of the water molecules present in the cryo-EM structure were kept fixed. In addition, the missing residues 240–248 and 334–339 in chain A were modelled using Maestro, and the missing atoms in the palmitoyl-oleoyl-phosphatidylcholine (POPC) lipids were added back using the CHARMM-GUI membrane builder [4]. For the solvated system, TIP3P water molecules [6] and counterions (Na${}^{+}$, Cl${}^{-}$) were added to neutralize the system and to set the ionic strength to 0.10 M, same as in Ref. [2]. The water molecules were added so that there would be at least 15 Å between the protein and the simulation box boundaries.

The atomic coordinate data of the initial structure with the aforementioned preparation steps (protein.pdb) is provided, as well as of the solvated structure (protein_solv.pdb). In addition, the atomic coordinates of the system after equilibration (protein_solv_equil.pdb) is provided. The equilibrated structure was obtained by first minimizing the system for 10,000 steps with the backbone atoms and heavy atoms of the heme cofactors restrained with a force constant of 10 kcal/(mol × Å${}^{2}$). Then, the system was gradually heated from 10 to 300 K over 4 ns and kept at 300 K for 6 ns in the NVT ensemble (using a Langevin thermostat with a friction coefficient γ of 5.0 ps${}^{-1}$) with the backbone atoms and heavy atoms of the heme cofactors restrained with a force constant of 1.0 kcal/(mol × Å${}^{2}$). Finally, the system was equilibrated for 10 ns in the NPT ensemble at 300 K and 1 bar (using a Langevin thermostat with a friction coefficient γ of 1.0 ps${}^{-1}$ and a Berendsen barostat with a time constant τ of 1.0 ps) with the backbone atoms and heavy atoms of the heme cofactors restrained with a force constant of 0.1 kcal/(mol × Å${}^{2}$). The root-mean-square deviation (RMSD) of all non-hydrogen atoms from the starting structure for the equilibration run plateaued during the equilibration run. All of these steps were executed using the Amber20 MD software package [7]. The Amber LEaP script that builds the initial dry and the solvated structures (tleap.in) is provided in this publication. This script uses the Amber ff14SB force field [8] for the standard residues, TIP3P water molecules [6], Joung/Cheatham ion parameters [9] for the counterions (Na${}^{+}$, Cl${}^{-}$), Generalized Amber force field (GAFF) [10] for ubiquinone, Amber Lipid 14 force field [11] for the POPC lipids, and the force field parameters for non-standard residues taken from Ref. [12] (heme.frcmod) or derived using MCPB.py [13] (mcpbpy.frcmod). More details for the MCPB.py derivation will be provided in the following sections.

### 2.2 Geometry optimization

Geometry optimizations were performed on the three heme cofactors (heme b${}_{558}$, heme b${}_{595}$, and heme d) using Gaussian 16 [14]. For all three heme cofactors, the side chains of the axial amino acid ligands were retained and the C${}_{\alpha }$ atom was turned into a methyl group. The C${}_{\alpha }$ and C${}_{\beta }$ atoms were kept fixed to preserve the axial ligands’ placement within the protein. For heme b${}_{595}$, four carbons in the pyrrole rings were also fixed. This was done because heme b${}_{595}$ has only one axial ligand.

We followed the *as isolated* mixed valence A${}^{1}$ state of the enzyme from Fig. S10 of Ref. [2] to determine the charges and multiplicities of the three heme cofactors. All of the propionates, except for the one in heme d, were protonated during the geometry optimizations so that the partial charges for all atoms, including the hydrogen in the hydroxyl group, could be obtained for the propionates. When making the final structure files, however, all of the propionates, except for one of the propionates of heme b${}_{558}$, were deprotonated since they coordinate with positively charged residues nearby in the cryo-EM structure [2]. Heme b${}_{558}$ is a low-spin heme (Fe${}^{3+}$) with neutral axial ligands (Met 393.A and His 186.A), so its overall charge was 0 and spin multiplicity was 2. Heme b${}_{595}$ is high-spin heme (Fe${}^{3+}$) with a deprotonated (thus negatively charged -1) coordinating axial ligand (Glu 445.A), making the overall charge -2 and spin multiplicity 6. While transitioning through state A${}^{1}$, heme d releases a water molecule, gets reduced by an internal electron redistribution, and quickly binds a dioxygen O${}_{2}$ molecule. We modelled the A${}^{1}$ state without a bound dioxygen molecule. The unbound state contains a high-spin heme Fe${}^{2+}$, and is the fully reduced state of the enzyme, i.e., one step in the reaction cycle before dioxygen coordinates to heme d to form state A${}^{1}$ (not shown in Fig. S10 of Ref. [2]). This unbound state has a -1 charge and a spin multiplicity of 5.

The initial geometry optimizations for heme b${}_{558}$, heme b${}_{595}$ and heme d were performed with the BP86 functional and the Los Alamos National Laboratory effective core potential double zeta basis set (LANL2DZ) [15] for the iron and Ahlrichs (Karlsruhe) split valence with polarization Gaussian basis set (def2-SVP) [16] for the rest of the atoms. Density fitting was also used for the initial geometry optimization to speed up the calculations [17]. Then, each system was submitted to another geometry optimization with the B3LYP functional with the same basis sets. After this, another round of optimization was performed with the Ahlrichs (Karlsruhe) valence triple-zeta with polarization basis set (def2-TZVP) for all atoms. The final optimization was done with the same B3LYP functional with def2-TZVP basis set with polarizable continuum solvent (PCM) [18], [19] at dielectric constant of 4; this dielectric constant is common for most proteins and enzymes. All these calculations contained GD3BJ empirical dispersion [20].

Gaussian calculations were executed using the Comet supercomputer at the San Diego Supercomputer Center (SDSC), part of the Extreme Science and Engineering Discovery Environment (XSEDE) [21]. As a result, heme b${}_{595}$ and heme d became more planar (with a dihedral angle of -1.2${}^{\circ }$ and -1.3${}^{\circ }$, respectively, formed by the central nitrogen atoms in each heme in the final optimized structures) compared to the initial cryo-EM structure (-3.0${}^{\circ }$ and -2.5${}^{\circ }$, respectively), in agreement with our expectations. Heme b${}_{558}$ retained its planarity (dihedral angle of 0.1${}^{\circ }$) in comparison to the experimental structure (dihedral angle value of 0.0${}^{\circ }$). The coordinate data for these geometry optimized structures are provided in this publication (heme_b558.xyz, heme_b595.xyz, heme_d.xyz).

### 2.3 Derivation of new force field parameters

The force field parameters solely pertaining to heme b${}_{558}$, heme b${}_{595}$, and heme d except for the spirolactone (γ-lactone) ring part were taken from Ref. [12] and Ref. [22], which report general force field parameters for heme cofactors and for heme b in cytochrome *c* oxidase, respectively. Heme d, unlike the other two heme cofactors, contains an unusual spirolactone ring and parameters for this part of the porphyrin ring were generated using the Antechamber package with GAFF parameters in Amber20 [23], as was done in Ref. [24]. These force field parameters for the heme cofactors, along with parameters generated with GAFF, are listed in heme.frcmod.

The key parameters involving the metal ions were obtained with the Python-based metal center parameter builder (MCPB.py) [13] and are listed in mcpbpy.frcmod. Since MCPB.py requires geometry optimization and force constant calculation of the atoms involved with the metal center, we used the B3LYP functional, LANL2DZ basis set for the iron, def2-SVP basis set for the rest of the atoms, and the implicit solvent model PCM with a dielectric constant of 4.0. The input structures were taken from the final geometry optimizations described in the previous section. In the force field parameters predicted by MCPB.py, all bond force constants between the iron and its ligating atoms were smaller than 200 kcal/(mol × Å${}^{2}$) and the equilibrium bond distances were smaller than 2.8 Å. Most angle force constants involving the iron were smaller than 100 kcal/(mol × radian${}^{2}$) with the equilibrium angle values greater than 100${}^{\circ }$, except for several of them for heme b${}_{558}$ (force constants greater than 100 kcal/(mol × radian${}^{2}$ or equilibrium angle values less than 100${}^{\circ }$) and a few for heme b${}_{595}$ and heme d (equilibrium angle values less than 100${}^{\circ }$). The potential barriers were zero for all of the dihedrals involving the iron. The Lennard-Jones radius obtained for the iron was greater than 1 Å for all three heme cofactors.

### 2.4 Point charges derivation

The atomic charges of the three heme cofactors and their axial ligands were derived from the structures obtained at the final geometry optimizations described in the previous subsection. The point charges were also computed at the same level of theory. We computed the electrostatic potential (ESP) on a molecular surface grid using the Merz-Singh-Kollman scheme [25], [26] in Gaussian. The ESP output was then used to generate restrained electrostatic potential (RESP) charges using Amber20’s Antechamber package [23]. The RESP charge calculation and fitting step was part of MCPB.py. We observed that the RESP charges of the metal ions in each heme were smaller than their oxidation state, as expected [27]. Since the axial ligands have charges different from standard Amber ff14SB charges, the axial ligands were incorporated as part of the appropriate heme cofactor system (HB1 for heme b${}_{558}$, HB2 for heme b${}_{595}$, HDD for heme d) and their backbones were separated out as different residues (HIO for histidine, MTO for methionine, GUO for glutamic acid) as done in Ref. [28]. Note that the propionates in the heme cofactors are not included, i.e., they are not part of the residues HB1, HB2, and HDD, and instead are separated out as different residues (PRP for protonated propionate and PRN for deprotonated propionate). The parametrization for the residues PRP and PRN were taken from Ref. [28]. Finally, in order to make each heme cofactor system equal to an integer charge, the infinitesimal remaining charge was distributed to all carbon atoms in each system. All of these residues are listed in heme.lib.

### 2.5 Setting up your own system

In order to set up the Amber input files of your own system with the novel heme parametrization reported in this work, users can repeat the same steps that we did for *Escherichia coli (E. coli)*, which we summarize next. First, we prepared the PDB file, starting from the one publicly available under the ID 6RKO at the Protein Data Bank, by modelling the missing residues and arranging the heme cofactors with the axial ligands, propionates, and axial ligands’ backbones (HB1, HB2, HDD, PRN, PRP, HIO, MTO, and GUO) renamed and/or redistributed accordingly to the description in the previous subsection. This PDB file is available as part of this publication: 6rko_final.pdb. Next, we run the Amber LEaP script (tleap.in), which loads the new force field parameters for the heme cofactor systems (heme.lib, heme.frcmod, mcpbpy.frcmod) and GAFF parameters for ubiquinone (ubiquinone.prepi, ubiquinone.frcmod), and generates the topology and coordinates Amber’s input file. As one can see in the tleap.in file provided in this publication, it is necessary that the user explicitly specifies the bonds between the propionates and the heme cofactor systems (HB1, HB2 and HDD) and the bonds between these heme cofactor systems with the backbone atoms of the axial ligands (HIO, MTO, and GUO). The Amber LEaP script creates the structure and topology files for the dry (protein.pdb, protein.prmtop, protein.rst7) and solvated (protein_solv.pdb, protein_solv.prmtop, protein_solv.rst7) systems. In case the user would like to change the protonation state of a given propionate residue, this can be done by simply modifying the residue name of this residue in 6rko_final.pdb from PRN to PRP, or vice-versa; PRP corresponds to the protonated propionate, and PRN the deprotonated propionate.

## CRediT authorship contribution statement

**Surl-Hee Ahn:** Conceptualization, Investigation, Data curation, Writing – original draft. **Christian Seitz:** Investigation, Data curation, Writing – review & editing. **Vinícius Wilian D. Cruzeiro:** Methodology, Validation, Data curation, Writing – review & editing. **J. Andrew McCammon:** Writing – review & editing, Supervision, Funding acquisition. **Andreas W. Götz:** Methodology, Validation, Writing – review & editing, Supervision, Funding acquisition.

## Declaration of Competing Interest

The authors declare that they have no known competing financial interests or personal relationships that could have appeared to influence the work reported in this paper.

## Appendix A Supplementary materials

Supplementary Data S1 Supplementary Raw Research Data. This is open data under the CC BY license http://creativecommons.org/licenses/by/4.0/ Supplementary Data S1
